# Biomarker Discovery by Novel Sensors Based on Nanoproteomics Approaches

**DOI:** 10.3390/s120202284

**Published:** 2012-02-16

**Authors:** Noelia Dasilva, Paula Díez, Sergio Matarraz, María González-González, Sara Paradinas, Alberto Orfao, Manuel Fuentes

**Affiliations:** 1 Centro de Investigación del Cáncer/IBMCC (USAL/CSIC), Departamento de Medicina and Servicio General de Citometría, University of Salamanca, Salamanca 37007, Spain; E-Mails: noeliadf@usal.es (N.D.); pauladg@usal.es (P.D.); smats@usal.es (S.M.); mariagg@usal.es (M.G.-G.); orfao@usal.es (A.O.); 2 Departamento de Química Analítica, Facultad de Ciencias Químicas, University of Salamanca, Salamanca 37008, Spain; E-Mail: ssparadinas@usal.es

**Keywords:** biomarker, cancer, nanosensor, high-throughput techniques, microarray, proteomics

## Abstract

During the last years, proteomics has facilitated biomarker discovery by coupling high-throughput techniques with novel nanosensors. In the present review, we focus on the study of label-based and label-free detection systems, as well as nanotechnology approaches, indicating their advantages and applications in biomarker discovery. In addition, several disease biomarkers are shown in order to display the clinical importance of the improvement of sensitivity and selectivity by using nanoproteomics approaches as novel sensors.

## 1. Introduction

Over the last decade, new-generation high-throughput (HT) methods have emerged and expanded in the field of proteomics, including next-generation sequencing and mass spectrometry technologies, which have enabled the study of increasing amounts of proteins with less sample requirements. Overall, this has translated into the possibility of performing multi-level studies of human diseases from the perspectives of genomics, transcriptomics and proteomics [1].

Proteomics research in human pathology has focused on the development of clinical applications for accurate diagnosis, early detection and prognostic assessment of human disease due to its potential utility in the identification of candidate biomarkers associated to disease status. Noteworthy, the elucidation of drugs’ mechanisms of action by these approaches might lead to further characterization of new therapeutic targets. Hence, one of the most relevant applications of clinical proteomics is the identification and characterization of extremely-low abundance metabolites that might be disease-specific or even prognostic-associated. Therefore, the identification of biomarkers represents the ultimate tool for the improvement of early diagnostics, patient monitoring and for the evaluation of the safety and efficacy of therapeutic strategies [2,3].

Consequently the detection of such low-abundance biomarkers in biological fluids (e.g., blood, urine or saliva) requires HT detection techniques. In this sense, the integration of nanotechniques and proteomics has led to the development of nanoproteomics, which provides a robust analytical platform for real-time and sensitive detection of low-abundance proteins [4–6].

Therefore, nanoproteomics offers a real-time multiplexed analysis performed in a miniaturized assay, with low sample consumption and high sensitivity, thereby finding an increasing number of potential applications in research. Quantum dots, gold nanoparticles, carbon nanotubes and nanowires are few nanomaterials which have demonstrated potential to overcome the challenges of sensitivity faced by conventional proteomics for biomarker detection [7]. However, concerns regarding the toxicity and biocompatibility of nanotechniques still remain to be explored and much work is being carried out to ensure their safety for biological applications [8].

In this manuscript, we briefly describe the applications of nanoproteomics for biomarker discovery in various diseases focusing on neoplastic processes and also on auto-immune, metabolic and infectious diseases.

## 2. Proteomics Technologies for Biomarker Discovery

The advancement in proteomics techniques has provided a useful platform for the discovery of potential disease biomarkers, being protein microarrays one of the proteomics platforms involved jn biomarker discovery. Protein microarrays are miniaturized and parallelized array technology approaches for protein-protein interaction analysis and protein profiling [9,10]. Typically, thousands of proteins are printed and immobilized on functionalized glass slides, which can be simultaneously studied and analyzed in a HT fashion, thereby offering a high potential for characterizing the biology of a given cell of interest. To date, a number of microarray formats have been developed and recently implemented; all of them have tested as a versatile platform for many diverse applications [11]. Between them, there are DNA-microarrays or protein-chips which can use nanoporous alumina as substrate [12].

Together with the advances in microarray technologies, increasingly sensitive and reliable detection methodologies are being currently developed [4]. Such protein detection systems have progressively undergone a relevant transition from label-based to more sensitive label-free technologies.

In general, label-based systems are mainly focused on the use of specific tags for target molecules as conventional fluorescent dyes and radioisotopes. In addition to the conventional labeling strategies other molecules are being gradually introduced for the improvement of detection methods such as quantum dots (QDs), gold nanoparticles (AuNPs) and carbon nanotubes (CNTs), among others. Moreover, dye-bead-based techniques such as flow-cytometry and magnetic bead-based detection strategies are currently being explored with promising results [4].

### 2.1. Label-Based Detection Methods for Biomarker Discovery

**Figure 1.**
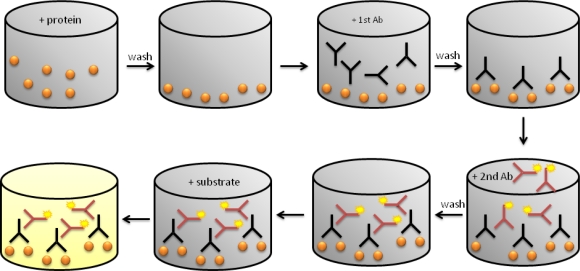
Schematic description of ELISA experiments.

Enzyme-linked immunosorbent assay (ELISA) is the label-based reference method to identify and quantify biomarkers (Figure 1). However, this technique has some disadvantages: (*i*) only one biomarker is measured per assay; (*ii*) 5 to 7 h are necessary to obtain the results; (*iii*) the valid range is only three orders of magnitude; (*iv*) two accurate antibodies are necessary. That is why in the last few years some variations on the classic ELISA have been appeared. Some examples are MesoScale^®^, Searchlight^®^, FASTQuant^®^. These alternatives have reduced the final volumes needed from 200 μL to 50–100 μL, as well as the time to three or four hours. Also, they can analyze 24 samples per assay and the range of detection has risen to four orders of magnitude [13].

In this sense, protein microarrays have been employed as an alternative of conventional ELISA procedures, because of HT characteristics. Miller *et al*. have used antibody microarrays for discovery of serum biomarkers in prostate cancer, the most common solid organ malignancy affecting men [14]. The high-density antibody microarray containing 184 antibodies enabled the successful identification of five potential protein biomarkers; von Willebrand factor, IgM, α1-antic-hymotrypsin, villin, and IgG for prostate cancer.

In this regard, cyanine dyes (e.g., Cy3 and Cy5) are among the most common fluorochromes employed for protein microarray detection because of their brightness and the reduced complexity of labeling proteins with charged lysine residues. Srivastava *et al*. used conventional Cy3 and Cy5 fluorophores for the identification of serum protein profiles in cystic fibrosis [15]. Zhou *et al*. developed a innovative two-color protocol for the detection of different labeled proteins from parallel serum samples immobilized on antibody microarrays, in which detection limits are below femtomolar protein concentration [16].

During the last years, additional assays (for simultaneous detection of multiple analytes) have been developed based on multiplexed capture antibodies. For example, a recently developed approach allows detection of the highest number of proteins [17]. This novel platform is based on protein-G coated suspension beads array composed of about 300 unique particle subsets; in each subset antibodies are subsequently immobilized and properly orientated. In addition, this platform incorporates another analytical dimension, which is protein fractionation by size exclusion chromatography from plasma mononuclear cells and/or cell lines. The analysis of the results obtained for this two dimensional platform provides information about complex elution profiles and illustrates its potential in large scale protein complex identification; opening a new approach for assessment of molecular interactions studies [16]. Moreover bead-based arrays can also be used for the kinetic characterization as well as interaction of enzyme with multiple substrates in a multiplexed analysis [18].

Despite many different label-based methods detect variation in optical properties of fluorochromes or tags; notwithstanding other label-based methods have been developed on stable isotopic labeling in order to avoid problems expected with fluorochrome probes and their efficient conjugation with biomolecules, for example: fluorochrome-protein conjugates cannot affect specific groups of the antibody binding, binding procedures may decrease the fluorescence of fluorochromes such that the conjugate only show 15–30% of the fluorescence corresponding to a free dye, working pH (fluorescein at pH 8 or rodamine at pH 7) may also affect the results.

**Figure 2.**
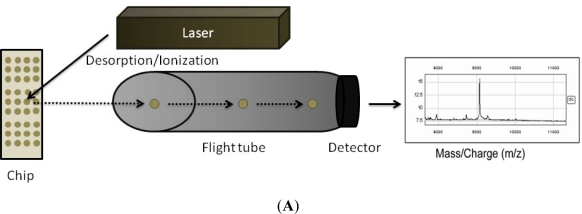

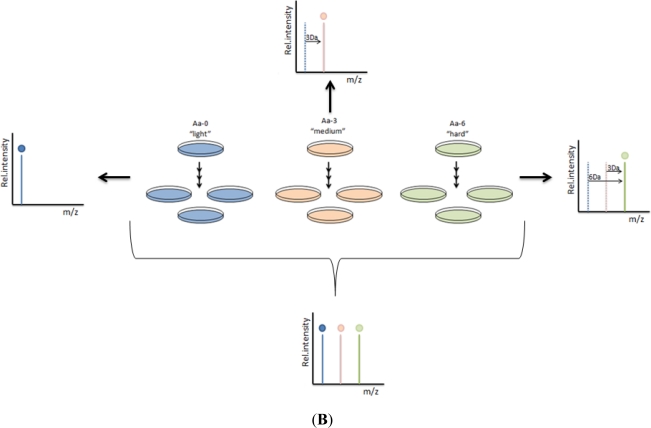
Schematic description of MS approaches used in biomarkers discovery. (**A**) SELDI-TOF; (**B**) SILAC.

Stable isotope labeling with aminoacids in cell culture (SILAC) is a label technique which is analyzed by mass spectrometry (Figure 2). It is based on the metabolic incorporation of non-radioactive heavy isotopic forms of amino acids into the cellular proteins while cells are growing.

SILAC allows identifying cell surface proteins which are expressed in a different amount according to state, studying the protein-protein interaction, the identification of tyrosine kinase substrates or the membrane proteins and the temporal dynamics through SILAC for 5-plexing [19]. For example, isotope labeling has been successfully used for differential proteomics studies between normal and tumorogenic cells. By this approach, it has been found at least a 2-fold up-regulation of vimentin, ATP synthase, and α-tubulin in prostate cancer cells exhibiting high tumorigenicity as compared with poorly tumorigenic cells. Their results suggest that these proteins may also be implicated in metastasis, showing SILAC ratios of 9.55, 5.92, and 2.17, respectively [20].

Waanders and collaborators have measured the signaling protein Growth factor Receptor-Bound protein 2 (Grb2), which is involved in signaling pathways. It is usually related to the Ras activation because of its SH2 domains, which allow it binding to phosphorylated tyrosins of kinase receptor, and SH3 domains, which allow it joining to the Ras-guanine exchange factor SOS [21].

### 2.2. Label-Free Detection Methods for Biomarker Discovery

Despite the wide use of the label-based techniques, researchers are increasingly using label-free techniques because they are cleaner, faster and simpler [22], but also because they usually are compatible with real-time detection [23].

There are several label-free methods, but all of them include three basic steps: (1) Sample preparation: protein extraction, reduction, alkylation and digestion; (2) Sample separation by liquid chromatography (LC or LC/LC) and analysis by tandem mass spectrometry (MS/MS); and (3) Data analysis including peptide/protein identification, quantification, and statistical analysis [22].

The most common label-free techniques widely used in proteomics, briefly described, are:

- Relative quantification by peak intensity of LC-MS: based on the lineal correlation between the area of the peaks in the LC-MS and the protein concentration.
- Relative quantification by spectral count: in these methods, protein quantification is accomplished by comparing the number of identified MS/MS spectra from the same protein in each of the multiple LCMS/MS or LC/LC-MS/MS databases.
- Absolute label-free quantification: it is used in the determination of absolute abundance proteins. This method gives the Protein Abundance Index (PAI), which is the number of identified peptides divided by the number of the theoretically observable tryptic peptides for each protein.

In general, the label-free techniques are proved to be useful for the study of real-time kinetics of biomolecular interactions, which are not hindered by interaction with tag molecules.

At present, there are many label-free detection strategies, such as surface plasmon resonance (SPR), CNTs, microelectromechanical cantilevers, surface-enhanced laser desorption ionization (SELDI)-time of flight (TOF)-MS, microfluidic purification chips (MPC), immunosensors based on channels of mesoporous silica (MPS), functionalized nanopipette probes, nanostructured electromechanical immunosensors featuring single-wall nanotubes (SWNT) forest and AuNPs [4,24–27]. Also, label-free protein-protein interactions were recently monitored using self-assembling protein arrays (named NAPPA microarrays) and atomic force microscopy (AFM), nanogravimetry, mass spectrometry and anodic porous alumina with the purpose of controlling the proteome alteration associated with cell proliferation, differentiation and neoplastic transformation [28–34].

**Figure 3.**
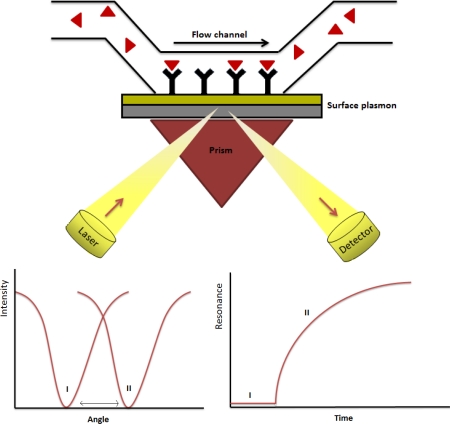
Schematic description of Surface Plasmon Resonance.

Among other label-free techniques, SPR is a detection technique which analyzes molecular interactions onto a planar surface, based on the generation of surface plasmons (Figure 3). These are oscillations of free electrons that propagate in parallel to a metal/dielectric interface, which allow measuring changes in refractive index close to the sensor surface [35]. SPR enables accurate determination of kinetic parameters (association to dissociation rate) of the binding process between molecules as well as evaluate the strength of the binding and the specificity of the occurring interactions on large scale. As a consequence, it is possible to measure bimolecular interactions in real-time with a very high sensitivity [36].

Currently, SPR has been coupled with imaging to give the surface plasmon resonance imaging methods (SPRi). SPR*i* can analyze hundreds of samples on a single array. It is possible to use whatever biomolecule and the probe molecule is immobilized onto a metal coated slide (commonly a gold thin layer: <50 nm). This technique is also based on the formation of surface plasmons. The polarized light is reflected depending on the interactions on the array and is collected to give an image. Ladd *et al*. made use of SPR*i* techniques for the detection of candidate diagnostic biomarkers in cancer using antibody arrays. Interestingly, SPR*i* is showing to be a potential useful technique for biomarker characterization in serum proteomic studies [37]. Noteworthy, additional studies have been reported the combination of SPR*i* with a microfluidic chamber to obtain continuous flow of the analyte during the experiment.

Finally, it is necessary to put emphasis on MPC, which was the first label-free system that used physiologic solutions, by detecting two biomarkers from a 10 μL sample of whole blood in less than 20 min [24].

### 2.3. Nanotechnology in Proteomics

Recently, there has been a great interest in applying nanomaterial-based electrochemical biosensors for the sensitive detection of biomolecules [38]. During the past few years, the potential of nanotechniques and nanomaterials in biomarker discovery has been studied [4,5]. Such emerging approaches are advantageous due to their high sensitivity, minimum sample requirements, accuracy, real-time sensing, and simplicity of the instruments, low cost and potential HT applications. In summary, nanotechniques offer several advantages with respect to classic proteomic techniques such as the miniaturization with a low amount of sample which leads to a higher sensibility and easier protocols.

Nanoparticles show highly selective protein absorption and they can reach subcellular locations, which has a great impact on protein interactions and cellular behaviour.

**Figure 4.**
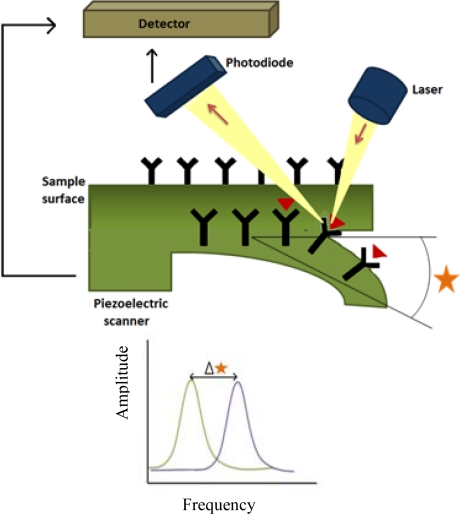
Schematic description of microcantilevers detection systems used in biomarkers discovery.

Among other nanomaterials, QDs, AuNPs, CNTs and silicon nanowires are promising candidates for biomarker detection and discovery. In addition, there are other promising nanotechniques which include microcantilevers (Figure 4), microfluidics, gold nanowires or silver nanomechanical resonators [8]. The technological aspects and working principles of commonly used nanoproteomics techniques for biomarker discovery have been discussed in detail in other reviews [4,5]. On the other hand, QDs, semiconductor nanocrystals, are applicable for labeling biomolecules and present advantages compared with organic dyes [4], such as brighter fluorescence and photo-stability. Finally, CNTs have shown higher sensitivity than standard ELISA, providing detection limits superior to this classical technique [8].

#### 2.3.1. Gold Nanoparticles

AuNPs can be modified with simple organic capping reagents or with high molecular weight biomolecules. Their unique optical properties, as well as their high thermal and electrical conductivity, make these materials valuable as components of biosensors, *in vitro* cell imaging and *in vivo* imaging and therapy [39].

Among metal nanoparticles, AuNPs have immense potential for cancer diagnosis and therapy on account of their SPR enhanced light scattering and absorption. AuNPs, which have to be labeled with accurate biomolecules, present a deviation in emission spectrum of scattered light because of effective binding of the analyte of interest from a protein sample by specific biomolecular interactions. This approach has been successfully used in PSA detection [40].

#### 2.3.2. Quantum Dots

QDs are semiconductors nanocrystals that exhibit unique electro-chemiluminiscent properties, strong light absorbance, bright fluorescence, size-tunable narrow emission spectra and provide excellent fluorescence quantum yields [4]. They are composed by elements from groups II–VI, III–V, or IV–VI of the periodic table, which can be attached to antibodies, aptamers, oligonucleotides, or peptides to be used to target cancer markers. These nanoparticles have many advantages such as their low toxicity, their biocompatibility, high quantum yields, diverse surface modification flexibility and they are used with different wavelengths of emission allowing the concurrent analysis of multiple biomarkers [41].

QDs are applicable for labeling of biomolecules such as peptides, proteins or oligonucleotides and considered as an attractive alternative of traditional organic dyes [4]. They can be employed to quantify biomarkers in assays based on fluorescence resonance energy transfer (FRET) or as acceptors in bioluminescence resonance energy transfer (BRET). QDs are bound to different antibodies and can label HER-2, which over-expresses on some human breast cancer and is quantified through FRET *in vitro* assays. But also, they are used as contrast agents for *in-vivo* cancer imaging and detection, for example in prostate cancer [41].

These nanoparticles have been used as biological probes for the simultaneous detection of multiple biomarkers directly from biological components [42]. During past years, several groups have reported the use of QDs for detection of different types of cancers. QD-antibody conjugates are also well suited for the multiplexing capabilities of semiconductor QDs, enabled the authors to detect four protein biomarkers (CD15, CD30, CD45 and Pax5) of Hodgkin’s lymphoma from lymphoma tissues [8].

#### 2.3.3. Carbon Nanotubes

Electronic bio-detection methods are rapidly emerging in diagnostics due to the technological advantages associated with sensitivity, signal amplification, low sample consumption, detection time and multiplexing capacity. CNTs have a high potential as electronic biosensors owing to their intrinsic electrical, thermal and spectroscopic properties [43]. Hence, CNTs are rapidly being adapted in clinical research and have shown considerable promise in cancer diagnosis and therapy. Furthermore, they have shown higher sensitivity than standard ELISA, providing detection limits superior to this classical technique [8].

Malthotra *et al*. constructed an electrochemical immunosensor using CNT arrays. They used secondary antibodies (HRP-labeled) for detection of low levels of IL-6 in experimental head and neck squamous cells carcionama cell lines [44]. CNTs have also been used as oxidase, dehydrogenase, peroxidase and catalase biosensors [45]. The use of CNT molecular wires offer great promise for achieving efficient electron transfer from electrode surfaces to the redox sites of enzymes. Better control of the chemical and physical properties of carbon nanotubes should lead to more efficient electrical sensing devices.

#### 2.3.4. Nanoparticle Biomarker Capture Technology

Recently, a new strategy has been developed for the rapid detection of target protein biomarkers by MALDI-TOF mass spectrometry. The approach relies on selective sequestering of target proteins from complex media by engineered microgels, which select proteins by their size (<30 kDa) and isoelectric points (protein pI < 6.5). In this case, protein extraction is not necessary [46]. Also, smart hydrogel particles have been developed in order to detect biomarkers present at low concentrations. With this purpose, an affinity bait molecule has been introduced into N-isopropylacrylamide (NIPAm particles). This structure is capable of performing three independent functions within minutes, in one step, in solution: (a) molecular size sieving; (b) affinity capture of all solution phase target molecules; and (c) complete protection of harvested proteins from enzymatic degradation [3,47].

#### 2.3.5. Nanocomposite Matrices for Sensors

Nanocomposite matrices, characterized by the presence of at least one component with two or three dimensions of less than 100 nanometers, are a mixture of inorganic, organic and biological materials. One recently described example is the mixture of cytochrome P450 with anodic porous alumina. In fact, a cytochrome P450 thin film developed and characterised in order to be used as cholesterol biosensor, is the most succesful inorganic biosensor based on P450ssc and anodic porous alumina, and will be explained in detail in the Biomarker Discovery in Metabolic Diseases section [29,48]. On the other hand, nanocomposites have been successfully employed as matrices suitable for protein microarrays. Nucleic Acid Programmable Protein Arrays (NAPPA) have been combined with anodic porous alumina (APA); and a few macromolecules have been successfully detected by this technique [48].

Nanocomposites have also been combined with multiwalled carbon nanotubes (MWNTs) providing a new material for conductometric acid vapours sensors [49]. In this way, carbon nanotubes can be introduced in conduction polymers, which allow biosensors with enhanced chemical and physical properties, and conduction polymers can be placed onto carbon nanotubes arrays [49].

## 3. Biomarker Discovery in Cancer

As was described in the Introduction section, protein biomarkers (see Table 1) can be used to define a kind of cancer, the stage of the disease or select a treatment [3,50].

Because of modern life style factors (sedentarism, nutritional habits, environmental contamination or life expectancy) some cancers are more prevalent, why it is necessary to find new biomarkers of the early stages to have more possibilities of earlier diagnostic of cancers [3,51]. Extracellular matrix proteins and elements secreted by a tumor can be diagnostic biomarker candidates. Secreted proteins are responsible for cell communication, so translating these signals into information could provide knowledge of the molecular mechanisms of neoplasia [51]. Also, modifications in glycosylation and the carbohydrate structure of proteins have been associated to cancer [52].

In the case of prostate cancer, Prostate Specific Antigen (PSA) is the biomarker usually used in the diagnostic of this pathology. PSA appears preferentially in the prostate, but it is produced by other tissues. Although it is a substance which is found in prostate, in patients it is localized at low concentrations in blood which are measured to make the diagnosis and the prognostics of cancer [51]. However, it is well known that PSA is not a biomarker as specific as it is necessary because the increase in PSA levels detected by 2D electrophoresis (2-DE) MALDI-TOF MS or SELDI Quadrupole-TOF (SELDI-qTOF) (Figure 3(A)) can be due to the age or prostatitis [52]. For this reason new biomarkers are needed [51].

Both prostatic acid phosphatase (PAP) and progastricsin (PG), which are overexpressed in prostate carcinoma, have been detected by 2-DE MALDI-TOF-MS. This technique has also identified a new potential biomarker: urinary calgranulin B/MRP-14. SELDI-MS has allowed detecting prostate cancer-24 protein, which appeared in 94% of prostate carcinomas and does not in normal cells. One of the most interesting lines which are being recently studied is likely biomarkers in prostatasomes, membranous vesicles secreted by the prostatic gland whose function is related with sperm motility and protection against female immunity in fecundation. Although more than 440 prostatasomes proteins have been recognized and categorized by LC-Electrospray ionisation/Mass Spectrometry (LC-ESIMS/MS) coupled with a gas phase fractionation (GPF), it is too soon to propose some new biomarkers. Also, metabolomics have identified a huge number of metabolites as potential biomarkers such as sarcosine, which is likely to indicate the progression to metastasis [52].

Breast cancer is the most prevalent cancer in women and the first cause of death, mostly because of the distant metastases. For this reason, it is necessary to identify the early stage biomarkers [51]. The lack of serum biomarkers drives to a too late detection of cancer, when surgery is no longer possible and/or metastasis processes are presented. An early detection might be possible only through both invasive and non-invasive techniques. Nowadays, a premature diagnosis is achieved by regular mammographies [53].

In breast cancer, many different mutations have been found, most of them in proto-oncogenes and/or tumor suppressor genes such as BRCA1, BRCA2, HER2-neu, C-MYC, and Cyclin D-1. As a result, auto-antibodies have been detected against the mutated genes such as p53 or heat shock protein 60 and 90 (hsp). 2D-PAGE, ELISA or NAPPA arrays have been some of the technologies used to try to detect breast cancer auto-antibodies. However, antibodies are not likely to be accurate biomarkers, unless they are into account together [53].

The most widely used serum marker in breast cancer diagnostics is CA 15-3, which is a soluble form of the mucin MUC1, which is in turn a marker of breast cancer. MUC1 is usually placed in the apical membrane of normal secretory epithelium, when malignant transformation has happened, MUC1 is translocated to the external plasmatic membrane, where is susceptible of suffering proteolytic cleavage. As a result, it is found as a soluble antigen which is usually detected by immunoassays. Unfortunately, as MUC1 changes its glycosylation pattern during neoplastic transformation, so it cannot be used as an early breast cancer biomarker [53].

In connection with glycoprotein and cancer, in breast cancer as in so many others, there are alterations in glycoproteins. The most known example is the Human Epidermal Growth Factor Receptor 2 (HER2/neu), which is a trans-membrane glycoprotein and whose overexpression means the malignant transformation of the tumor [53].

Ovarian cancer, one of the most aggressive and lethal cancers in women, lacks of a non-invasive diagnostic exam in order to detect it in the earliest stages [51]. Comparing samples from patients with ovarian cancer and healthy individuals, it was found that CA125 had the sensitivity of 60.7% and the specificity of 55% for distinguishing ovarian cancer from non-cancer samples. Moreover, four proteins were found, which are better biomarkers than CA125, using SELDI-TOF-MS protein chip technology; which is widely used to monitor the patients after the chemotherapy [54].

Pancreatic ductal adenocarcinoma (PDAC) is another of the most aggressive cancers and the problem lies in the fast metastasis [51]. This cancer has the worst prognosis and the mortality percentage is very similar to the rate of incidence. The best biomarker in pancreatic cancer is CA 19-9, which is a sialylated Lewis antigen of the MUC1 protein and is detected by serum immunoassay [55]. Although sensitivity is about an 80% and specificity about 90%, this biomarker also appears in some diseases such as cirrhosis or chronic pancreatitis. That is why it cannot be used as an accurate biomarker. Most of the pancreatic cancers are discovered by computed tomography (CT) or magnetic resonance imaging [56].

Other kinds of cancer, for example colorectal or lung cancer, are not related with specific and accurate biomarkers because of problems such as low concentration or the masking by other proteins. Colorectal cancer is one of the most insidious cancers. The preferential treatment is surgery after neo-adjuvant treatment, but in most of cases metastases reappear some years later. Although biomarkers for metastasis are not known, researchers are making an effort to discover them. Lung cancer is the most prevalent and the major cause of death worldwide nowadays. Melanoma is a lower incidence dermatological cancer, but it is responsible of 80% of skin cancer death because of its fast metastasis to the brain [51].

## 4. Biomarker Discovery in Autoimmune Diseases

The importance of the detection of biomarkers for autoimmune diseases (see Table 2) lies in the need of an early detection of diseases, as well as the disease progression to disability and the response to therapy [57]. Autoimmune diseases appear in 3% of the population and until, now the diagnosis is made through clinical examination, laboratory tests and imaging techniques. Since last decade, biomarkers for diagnostic of immune diseases employing different proteomics approaches have been studied [58].

A specific characteristic of this disease is the presence of autoantibodies in systemic circulation as well as in specific proximal fluids and tissues. The main problem appears as a consequence of the immunity against self-molecules, auto-antigens, which can be related with the alterations on the gene which regulate the self-tolerance paths [57]. Proteomics allow the study of the key events which happen in the protein level such as post-translational modifications or antibody production [57].

In rheumatoid arthritis (RA), a systemic inflammatory disease related with alterations in human leukocyte antigen (HLA)-DRB1 locus, it has been necessary to find accurate biomarkers which identify the early stages of the disease, before cartilage damage ocurrs [59].

Diverse proteomic technologies have contributed to the discovery of biomarkers in autoimmune diseases, such as: (*i*) 2-DE and MS for auto-antigen discovery; (*ii*) autoantigen microarrays to typify autoantibody responses; (*iii*) antibody array technologies to profile cytokines and other biomolecules; (*iv*) reverse-phase protein arrays to analyze phosphoproteins; (*v*) flow cytometric analysis of phosphoproteins [57,60].

For example, Zhen and colleagues have developed microarrays which consist of putative and candidate genes printed by a robot over the array and probed against immune or control serum. The potential interaction is detected by fluorophore-conjugated anti-human secondary antibodies and they have found that the appearance of citrulline in RA means more severe disease and the detection of native and unmodified peptides is associated with mild disease [61].

Western blotting has allowed identifying some post-translational modifications variants of proteins have been characterized as auto-antigens such as citrullinated alpha-enolase in RA [57].

However, traditional MS or array-based proteomic assays face several limitations in the detection of multiple low abundance biomarkers from complex biological samples under clinically relevant conditions due to their sensitivity and specificity issues. Moreover, the detection process is very slow and it is often characterized by an unsuitable screening of large numbers of samples. These challenges of proteomics techniques prompted researcherd to apply different nanotechniques for biomarker discovery in auto-immune diseases.

Peptide-coated nanotubed are one of the recent approaches for the development of new immunosensors for diseases with specific serological autoantibodies, such as RA. Drouvalakis *et al*. determined cyclic citruline from patient serum in fentomolar (fM) range [62].

Wegener’s granulomatosis is a rare auto-immune disease coupled with anti-neutrophil antibodies, which affect blood vessels as well as various other organs. Proteinase 3 (PR3) is a potential serum biomarker for this autoimmune disease and is used for routine diagnosis of the disease. Although it is difficult to detect such a low abundance protein in complex samples, Chen and collaborators have developed a nanoproteomics approach for detection, at 1 fM level, of the target molecule by using antibodies conjugated with Raman tags for selective detection of PR3 [63]. In this case, the sensitivity which has been shown is higher than conventional fluorescence-based protein microarrays and traditional ELISA assays.

Biomarkers for systemic lupus erythematosus (SLE) and systemic sclerosis (SSc), both autoimmune connective tissue diseases, can be found using recombinant antibody microarrays. Carlsson and collaborators have developed a system in order to target mainly immunoregulatory proteins present in these autoimmune diseases. In this way, they found differentiation biomarkers between SLE and SSc. They also, observed differences increased with severity of SLE; thus, IL-2, IL-12 and IFN-γ were detected [64]. Hence, proteomics has shown to be a great candidate to detect disease biomarkers and control the phenotypic subsets and activity of diseases.

## 5. Biomarker Discovery in Infectious Diseases

Besides various cancers and autoimmune diseases, serum proteome analysis has also been tested for many infectious diseases such as tuberculosis, leprosy and hepatitis, among others [65,66].

Infectious diseases have become the leading cause of death in developing countries. That is one of the reasons why biomarkers (see Table 3) to achieve detection kits are needed [67]. New tools can help to identify the pathogen, evaluate the illness severity or establish the best treatment. Although lateral flow immunoassays, ELISA and the polymerase chain reaction (PCR) have been used with their limitations in the developed countries, these techniques frequently cannot be used in the developing countries. The World Health Organization has established the accurate characteristics to the diagnostic devices in the developing countries. They are summarized in the ASSURED criteria: A for Affordable, S for Sensitive, S for Specific, U for User-Friendly, R for Robust and Rapid, E for Equipment-Free and D for Deliverable to those who need them [68].

Among the potential biomarkers are products and targets with immunological memory of a pathogen, molecules which allow differentiating between infected and healthy individuals as well as assays, which recognize pathogen proteins and molecules. Until now, the main techniques used to detect the infectious individuals were serology and molecular methods. Although work on proteomic approaches is going on, the use of biomarkers will depend on our understanding of each infectious disease immunopathogenesis [67].

Agranoff and collaborators made use of SELDI-TOF-MS for identification of 20 most discriminatory proteins by comparing serum profiles from 179 tuberculosis subjects [69]. By MALDI-TOF-MS, both proteins amyloid A and transthyretin were demonstrated as potential serum biomarkers for early diagnosis of tuberculosis.

Another study identified differentially expressed proteins by MALDI-TOF and MALDI-TOF-MS/MS of leprosy patients and healthy individuals [70]. A significant increase in one of the isoforms of 2α chain of haptoglobin was determined in leprosy patients.

During the last years, several nanoproteomics studies have been conducted to study different types of infectious diseases. Tang *et al*. have demonstrated the selective detection of anthrax protective antigen from serum samples using a novel sensing approach based on europium nanoparticle-based immunoassay. This novel approach offered 100-fold enhancement in detection limit (0.01 ng/mL) as compared to the traditional colorimetric development reagents of ELISA assays [71].

Another recently described approach allows the detection of dengue virus infection, based on a combination of integrated microfluidic system and magnetic beads. The designed strategy reaches high sensitivity levels (21 pg) in a 30 min assay, indicating the potential of such sensing strategies for the development of rapid diagnostic test in infectious diseases [72].

Over the last two decades, anti-retroviral therapy (ART) has been successfully used reducing the morbidity and mortality of HIV-1. However, many patients have developed several immune abnormalities and their risk to suffer non-AIDS associated diseases has increased. Owing to that, it is necessary to find biomarkers which allow classifying patients into groups at risk of suffering non-AIDS diseases. HIV infected patients are also increasingly susceptible to suffering opportunistic pathogen infections, which is termed as immune restoration disease (IRD). One of the most frequent and severe IRDs is tuberculosis (TB). Usually, this combination highly increases the worsening of the pathology, particularly, the progression of extrapulmonary disease and lymphadenitis. Oliver and Price found in 2011 that CCL2 chemokine shows a decrease in its levels when a patient submitted to ART is going to develop TB [67,73].

Prion diseases, such as Creutzfeldt-Jacob (CJD), are neurodegenerative diseases related to the transformation of the normal host cellular prion protein (PrP^c^) into the abnormal protease-resistant isoform (PrP^Sc^). The traditional diagnosis is based on the detection of proteinase K resistant, misfolded form (PrPSc) of cellular prion protein in the central nervous system (CNS). Biomarkers are needed to detect the disease in the early stages to avoid the progression of the disease over time. Sanchez *et al*. found a 13.4 KDa protein in cerebrospinal fluid (CSF), which was analyzed by cationic exchange chromatography, sodium dodecyl sulfate-polyacrylamide gel electrophoresis (SDS-PAGE), and LC-MS/MS and it was revealed that the protein was cystatin C [74]. This protein had been found by other researchers, also in blood, and it is known that its increase in CJD affected patients is related with the disease. Mabbott *et al*. have found dendritic cells and macrophages carrying PrP^Sc^. Macrophages may even transport the abnormal protein in the absence of Follicular Dendritic Cells (FDCs) that is why the authors have considered the possibility that macrophages are a new structure in prion accumulation. On the other hand, dendritic cells can spread the infection towards other parts of the body [75].

## 6. Biomarker Discovery in Metabolic Diseases

Serum profiling has also provided biomarkers (see Table 4) for many other human diseases such as non-alcoholic fatty liver disease, diabetes, ischemic and hemorrhagic stroke [76]. Here, some of them are listed:

- Glucose biosensor: glucose levels can be monitored either *in vivo* or *in vitro*. Nowadays, there are biosensors based on conducting polymers, which have been shown to be useful for glucose estimation form 1 to 40 mM and a stability of about 6 days. A novel glucose biosensor based on MWNTs have been developed improving upon the previous ones [29].
- Lactate biosensor: until now, two different technologies have been approached for the development of nanosystems: film electrodes in combination with microdialysis systems and screen printed electrodes, which have shown a linear dynamic range from 0.2 to 1 mM of lactate and a stability of about 3 weeks.
- Urea and creatinine biosensors: most of them are based on detection of NH_4_^+^ or HCO_3_^−^ sensitive electrodes. A composite film of electropolymerized inactive polypyrrole and a poly ion complex has been developed.
- Cholesterol biosensor: the measurement of cholesterol is based on an amperometric biosensor. This sensor responds even in presence of potential electrical interferences, as l-ascorbic acid, pyruvic acid and uric acid. The most successful cholesterol biosensor, recently described, is the one based on the P450-linked side chain cleaving enzyme (P450ssc), which consists of P450 cytochrome and adrenadoxin, a P450 reductant, and it has been used to make an amperometric biosensor to detect and measure the LDL-cholesterol in liquid solution. It is based on the Anodic Porous Alumina (APA), which is a specific size porous matrix, and in the organic poly-cationic poly-l-Lysine (PLL), which allows a molecular anchorage as well as a direct electron transfer. The APA layer is placed onto a rhodium–graphite screen-printed electrode (s.p.e.) and the P450ssc was immobilized through the PLL. The enzyme and analyte binding leads to a redox reaction, which can be translated into an electrical signal producing a direct electron transfer between the enzyme and the electrode. The cholesterol detection and measurement is made by cyclic voltammetry (CV). It is achieved a very good stability mainly because the enzyme was very strongly trapped in the APA/PLL matrix [29,48].
- Uric acid biosensor: useful in gout, hyperuricaemia and Lesch-Nyhan syndrome.

Heart fatty acid-binding protein has been identified as a novel diagnostic serum biomarker for earlier diagnostic of stroke using a gel-based proteomic approach.

Serum proteomics have been also found to be a good alternative to liver biopsy for detection of common chronic liver diseases like non-alcoholic fatty liver disease. Recently, it has been described fibrinogen B chain, paraoxonase 1, prothrombin and serum amyloid P component as novel serum biomarkers [65] using a LC-MS/MS approach [76].

In 2007 Kim *et al*. identified extracellular glutathione peroxidase and apo-lipoprotein E as potential serum biomarkers using 2D ESI-qTOF MS/MS approach, and verified their results by Western blotting and ELISA in diabetes mellitus patients. This represents an alternative to conventional finger-prick capillary blood glucose self-monitoring, which has several disadvantages: it is painful, it cannot be performed when the patient is sleeping or doing some activity and it is intermittent, which means it can miss dangerous fluctuations in blood glucose concentrations between tests. For all these reasons, the ideal blood glucose monitoring would therefore be continuous and non-invasive [77].

Measurement problems in diabetes can be solved with nano-approaches, such as biocompatible nanofilms, glucose nanosensors, quantum dots or gold nanoparticles [78].

The detection of glucose levels used as diabetes biomarker, can be made through encapsulation of glucose sensors that could be implanted in the body avoiding degradation and denaturation maintaining, at the same time, glucose access and detectable signal change. This kind of encapsulation can be carried out by the electrostatic layer-by-layer (LBL) nanoassembly of capsules composed of multi-layers of polymer films [78]. Also, nanotechnology has increased the surface area of sensors. So far, sensors in diabetes are based on electrochemical enzymatic measurements with screenprinted eletrodes. However, nanotechnology can offer higher surface area/volume ratios as well as enhanced optical properties (QDs, AuNPs, SERS) allowing improvements in accuracy, size, lifetime and usability of sensors for the treatment of diabetes [79].

The principal strategy used in diabetes is based on standard enzymatic electrochemical detection of glucose. In this way, we can use CNTs, nanowire arrays fabricated from ruthenium and gold, which increase surface area and improve electrochemical detection. On the other hand, nanomaterials allow the development of direct oxidation glucose sensors as replacements to biological recognition sensors. For this purpose, it can be used porous films, nanorods and nanoparticles composed of silver, gold, nickel and nickel/palladium.

It is also possible to design nanomaterial-based sensors to detect glucose through changes in pH or charge, such as field effect transitor (FET), which seems to be a good option. Finally, for *in vivo* continuous monitoring, fluorescence-based sensors offer several advantages. In this case, sensors would be implanted into the skin of the patient. They would have to be replaced weekly or monthly because of problems with signal degradation, however with this strategy, it is not necessary to take blood samples [79].

During the last decade, emerging nanotechniques have been using for biomarkers detection in metabolic diseases. Lin *et al*. have reported simultaneous label-free electrochemical detection of two cardiovascular biomarker proteins, CRP and myeloperoxidase directly in human serum. In this nanoproteomics approach, high-density nanowells were prepared on top of each electrode using nanoporous silica membrane to improve sensitivity and selectivity (down to 1 pg/mL) [26].

## 7. Concluding Remarks

Proteomics research has revealed many novel disease biomarkers by applying various top-down and bottom-up approaches including gel-based techniques, MS, affinity separation and microarrays. Technological working aspects of different conventional proteomics techniques have been described in other reviews.

Despite the immense progress, biomarker discovery is still facing several biological and technological challenges such as the wide dynamic range of protein concentrations, difficulty of detection of low-abundance proteins and extreme variations between individuals.

During the last years, nanotechniques have undergone a significant progress for reliable handling the complexity of the cell proteome. Therefore a number of nanotechniques have been lately used for diverse applications such as biomarker discovery, label-free protein detection, study protein-protein interactions and printing protein microarrays. The advantages offered by these approaches have allowed to be successfully coupled with the rapidly expanding field of proteomics. Among other relevant emerging techniques, CNTs, QDs or AuNPs have drawn great attention due to their potential to minimize sample and reagent consumption.

However, nanotechniques still face several limitations to be resolved for widespread application in biomarker discovery. Currently, new proteomics and nanotechnology disciplines are being progressively adopted by clinical researchers due to the availability of multiple-novel techniques and all the potential applications to deep into the knowledge of the pathophysiology of unresolved diseases. All the methodologies and techniques briefly described in this minireview, might eventually lead to the characterization of new molecular entities and/or disease-associated molecular modifications for improving diagnostic and prognostic stratification. Despite this, many efforts are still required to implement the current status of these approaches towards clinical standardization.

Nowadays, it is possible to anticipate a significant development in the near future that will make nano-proteomics for biomarkers discovery field more robust, sensitive, reliable and above all, biocompatible and environmentally friendly.

## Figures and Tables

**Table 1.** A list of cancer biomarkers detected by novel sensors based on nanoproteomics approaches.

**CANCER**	**TUMOR BIOMARKER**	**DETECTION TECHNIQUES**
Breast cancer	BRCA1, BRCA2	Protein Truncation Test (PTT) and western blotting
	C-MYC	FISH
	CA 15.3, CEA, HER2/neu	Immunohistochemistry (ELISA)
	HER2/neu	Quantum dots (QD) and optofluidic ring resonator sensors
	CEA	Gold nanoparticles based techniques, quantum dots (QD) and silicon photonic microring resonators
	Auto-antibodies against p53 or heat shock protein 60 and 90 (hsp)	2D-PAGE, ELISA or NAPPA arrays
Colorectal cancer	CEA, CA 19.9, CA 72.4	Immunohistochemistry
	CEA	Gold nanoparticles based techniques, quantum dots (QD) and silicon photonic microring resonators
	ANXA3, BMP4, LCN2, SPARC, MMP7, MMP11	Immunoblotting and tissue microarray analysis
Epithelial neoplasia	CEA, CYFRA 21-1	Immunohistochemistry
Gastric cancer	CEA, CA 19.9	Immunohistochemistry
	CEA	Gold nanoparticles based techniques, quantum dots (QD) and silicon photonic microring resonators
Germ cell tumor	hCG, AFP, LDH	Immunohistochemistry
	AFP	Gold nanoparticles based techniques, quantum dots (QD), carbon nanotubes (CNTs)
Head and neck cancer	Desmoglein-3, Cytokeratin 4, Cytokeratin 16, Desmoplakin, Vimentin	RPLC-MS/MS: MS-count of unique peptides per protein
	Keratin 4, Keratin 13, Cornulin, Small proline-rich protein 3	2D DIGE
	14-3-3 sigma, 14-3-3 zeta/delta, hnRNPK, S100-A7, PTHA	iTRAQ
Hepatocarcinoma	AFP	Immunohistochemistry, gold nanoparticles based techniques, quantum dots (QD), carbon nanotubes (CNTs)
	Hsp27, Hsp70, and glucose-regulated protein 78	2-DE and MS/MS
Lung cancer	CA125	SELDI-TOF-MS
	HER2/neu, CYFRA 21-1, NSE, CEA	Immunohistochemistry (ELISA)
	HER2/neu	Quantum dots (QD) and optofluidic ring resonator sensors
	CEA	Gold nanoparticles based techniques, quantum dots (QD) and silicon photonic microring resonators
Lymphoma	LDH, β2-microglobulin	Immunohistochemistry (ELISA)
Myeloma	Ig, β2-microglobulin	Immunohistochemistry (ELISA)
Ovarian cancer	CA-125	SELDI-TOF-MS
	HER2/neu	Quantum dots (QD) and optofluidic ring resonator sensors
	LDH, CA 15.3, HER2/neu, CEA, CA 19.9	Immunohistochemistry
	CEA	Gold nanoparticles based techniques, quantum dots (QD) and silicon photonic microring resonators
	Tropomyosin family, actin family, triosephosphate isomerase family, Hsp60	Peptide fragment matching and MS/MS
Pancreatic cancer	CA 19.9, CA 72.4, MUC1	Immunohistochemistry (ELISA)
Papillary and follicular thyroid carcinoma	Thyroglobulin	Immunohistochemistry and PCR-RT
Prostate cancer	PSA	Gold nanoparticles based techniques, quantum dots (QD), carbon nanotubes (CNTs), silicon nanowires and 2D cantilever array chip
	PAP, PG, urinary calgranulin B/MRP-14	2-DE MALDI-TOF-MS
	Prostate cancer-24 protein	SELDI-MS
	HER2/neu	Quantum dots (QD) and optofluidic ring resonator sensors
Testicular cancer	AFP	Gold nanoparticles based techniques, quantum dots (QD), carbon nanotubes (CNTs)
	β-hCG	Immunohistochemistry
Trophoblastic disease	Gonadotropin	Immunohistochemistry

**Table 2.** A list of autoimmune biomarkers detected by novel sensors based on nanoproteomics approaches.

**AUTOINMUNE DISEASE**	**AUTOINMUNE BIOMARKER**	**DETECTION TECHNIQUES**
Diabetes	has	Magnetic relaxation nanosensors
Behcet’s disease	α-tropomyosin	IEC, SDS-PAGE and ESI-MS (sera)
	Selenium-binding protein	2-DE and immunoblot (sera)
	α- enolase, Haptoglobin	2-DE and MALDI-TOF-MS (sera)
	Serum amyloid A	2-DE and MALDI-TOF/TOF-MS (sera)
Juvenile idiopathic arthritis	Transferrin, ceruloplasmin, Serum amyloid A	Chromatographic protein chips, SELDI-TOF-MS (sera/urine).
	Serotransferin, GAPDH, α-1 anti-trypsin	IP of CIC’s, 2DE, ESI-MS/MS (serum)
	Citrulinated fibrinogen, complement 3, complement 1q	IP of CIC’s, SEC/LC, ESI-MS (serum)
	Complement 3c, apolipoprotein AII,vitamin D binding protein	DIGE, MALDI-TOF/TOF (plasma, synovial fluid)
Type 1 diabetes	GDC glutamate decarboxylase	Supramolecular protein nanoparticles
Rheumatoid arthritis	Cyclic citrulline peptide	ELISA and Peptide-coated nanotube-based biosensor
	peptides of C-reactive protein (PCR)	SDS-PAGE and triple quadrupole (TQ)-MS by multiple-reaction monitoring (MRM)
	p38 MAPK	Flow cytometry and Western blotting
	RF	ELISA
Wegener Granulomatosis	PR3	Carbon nanotubes as multicolor Raman labels

**Table 3.** A list of infectious biomarkers detected by novel sensors based on nanoproteomics approaches.

**INFECTIOUS DISEASE**	**INFECTIOUS BIOMARKER**	**DETECTION TECHNIQUES**
Anthrax	Anthrax protective antigen	Europium nanoparticlebased immunoassay
Anthrax	*Bacillus anthracis* Protective antigen	Multichannel waveguides
Candidiasis	D-arabinitol	One step electrodeposition
Chronic liver diseases, cirrhosis and hepatocellular carcinoma	Hepatitis B and C virus antibodies	Nano-gold immunological amplification on protein chip
Diptheria	Diphtheria antigen	Potentiometric immunosensor
Food borne disease	Listeria monocytogenes	Bioconjugated silica nanoparticles probe with FITC
Food borne illness	Salmonella	Bioconjugated nanoparticles
Gonorrhea	*Neisseria gonorrhoeae*	Nano-structure zinc oxide film
Hepatitis B	HBV virus	Microfluidic device with microbead array and QD
HIV-1 Infection	HIV-1 p24 antigen	Nanoparticlebased immunoassay
	HIV-1 p24 Gag protein	Nanoparticle-based bio-barcode amplification
Parasitic disease	*Schistosoma japonicum* antibody	Silver-enhanced colloidal gold metalloimmunoassay
Salmonellosis	*Salmonella typhimurium* antigen	Hybrid electrochemical/magnetic assay
Tuberculosis	Protein amyloid A, transthyretin	Surface-enhanced laser desorption ionization time of flight (SELDI-TOF) mass spectrometry

**Table 4.** A list of metabolic biomarkers detected by novel sensors based on nanoproteomics approaches.

**METABOLIC DISEASE**	**METABOLIC BIOMARKER**	**DETECTION TECHNIQUES**
Diabetes mellitus	Glucose	Glucose sensors: electrostatic layer-by-layer (LBL) nanoassembly of capsules composed of multi-layers of polymer films, standard enzymatic electrochemical and nanomaterial-based sensors
	Extracellular glutathione peroxidase, apo-lipoprotein E	Spectrophotometry and electrochemical techniques
Gout	Uric acid	Spectrophotometry and electrochemical techniques
Hyperuricaemia	Uric acid	Spectrophotometry and electrochemical techniques
Lesch-Nyhan syndrome	Uric acid	Spectrophotometry and electrochemical techniques
Chronic liver diseases	Fibrinogen B chain, paraoxonase 1, prothrombin, serum amyloid P component	Immunohistochemistry
